# Sero-epidemiology and associated risk factors of brucellosis among sheep and goat population in the south western Nepal: a comparative study

**DOI:** 10.1186/s12917-021-02835-8

**Published:** 2021-03-25

**Authors:** Tulsi Ram Gompo, Rubina Shah, Ishwari Tiwari, Yam Bahadur Gurung

**Affiliations:** 1 Central Veterinary Laboratory, Kathmandu, Nepal; 2 Institute of Agriculture and Animal Science, Tribhuvan University, Paklihawa, Nepal

**Keywords:** Brucellosis, Zoonotic disease, Nepal, Sheep and goat, Livelihood, Risk factors, One health approach

## Abstract

**Background:**

Brucellosis is a zoonotic disease caused by *Brucella* spp. In Nepal, the presence of brucellosis in small ruminants, namely sheep and goats, has impacted farmers’ livelihood and the food safety of consumers. A cross-sectional study was conducted in Rupandehi district of Nepal during January to March 2020 to investigate the seroepidemiology and associated risk factors of brucellosis in the sheep and goat population. Altogether, 19 sheep and 60 goat farms in the district were visited. Owners were interviewed to get information on animals, including their management and movement patterns. Three hundred fifty-seven samples (80 sheep and 277 goat samples) were collected proportionately based on farm sizes. Each serum sample was tested with Rose Bengal Test and ELISA to estimate the seropositivity of brucellosis. Logistic regression was carried out to calculate corresponding odds ratios of each variable associated with detection of brucellosis.

**Results:**

At the farm level, 31.6% (6/19; 95% CI: 12, 54%) of sheep farms and 3.3% (2/60, 95% CI: 0.9, 11.4%) of goat farms were seropositive to brucellosis. Out of 80 sheep serum samples, 12 (15%; 95% CI: 8.79–24.41%) and out of 277 goat serum samples, three (1.1%; 95% CI: 0.37–3.14%) were seropositive to brucellosis. Age greater than 1.5 years (OR = 5.56, 95% CI: 1.39, 29.38; *p* = 0.02) and herd size of greater than 100 (OR = 4.74, 95% CI: 1.23, 20.32, *p* = 0.03) were identified as significant risk factors for seropositivity of brucellosis in the sheep population. While in the goat population, none of the variables was identified as a significant risk factor.

**Conclusion:**

The study provides evidence that the older sheep and the sheep from the large herds were at higher risk of brucellosis. A control program should be put in place immediately in the sheep population because they may transmit infections to other livestock as they were regularly moved for grazing and selling purposes. Also, strict biosecurity measures should be implemented among pastoralists to prevent brucellosis transmission in them. We suggest further one health-based study to reveal the transmission dynamics of brucellosis between animals and humans.

## Background

Brucellosis is an economically important zoonotic disease caused by the gram-negative bacteria of *Brucella* species [1–3]. People contract Brucellosis by the consumption of unpasteurized dairy products, undercooked meat [4–6], occupational exposures through handling of aborted fetus or placenta of infected animals [4], and inhalation of contaminated aerosol during the processing of the animal products [4, 5, 7]. Brucellosis creates significant economic losses to the livestock industry worldwide because it usually results in abortion, infertility, and decreased milk and meat production. The disease has been successfully managed or eradicated from several developed countries, but it is still endemic in livestock and human populations in resource-poor countries [8].

Ruminants are highly susceptible to brucellosis compared to other domestic animals [9]. Brucellosis in small ruminants is largely caused by *B. melitensis* and *B. abortus*, with clinical manifestations such as abortion, retention of placenta, infertility, epididymitis and sometimes arthritis [10]. In small ruminants, mainly goats and sheep, the infected animals remain as the primary transmission source to their herds. Most *Brucella* species, except *B. ovis*, are considered pathogenic to humans as they carry a surface antigen of smooth lipopolysaccharide (S-LPS) involved in the virulence of these bacteria [11, 12].

Small ruminants are important contributors to the livelihood of Nepalese farmers and goat, in particular, is considered as the “poor man’s cow” [13, 14]. They are one of the principal commodities of the livestock production system in Nepal. There is an estimated population of 11 million goats and 0.8 million sheep in Nepal [15].

Nepalese sheep support the local carpet industry [16], while goat meat provides the second (20.36%) most substantial volume of meat for consumption after buffalo meat (54.34%) in Nepal [15]. The demand of goat meat is the highest during September to November every year, as the two biggest festivals, viz. Dashain and Tihar, fall within this period [17]. Import and rapid movement of small ruminants during festival seasons [18–20], potentially pose the highest risk of livestock disease transmission between the ruminant populations. There may also be an increased public health risk from diseases like brucellosis when people choose to slaughter goats and sheep at their homes for the festivals [21, 22]. Another risk associated with the infected sheep and goat flocks relates to the transhumant or nomadic form of migration, where livestock and pastoralist movement occurs between mountain pastures in warm seasons and lower altitudes the rest of the year. These activities increase the risk of disease transmission between livestock and humans [23].

Although there are some published studies describing the seroprevalence of animal and human brucellosis in Nepal [24–26], there are no studies that describe the risk factors related to animal brucellosis in Nepal. Identifying risk factors and implementing prevention and control programs could lead to a decrease in the disease burden in small ruminants, with consequent improvements in human health. We aimed to describe the comparative seroprevalence and risk factors of goat and sheep brucellosis together, as they are generally found in contiguous herds or mixed farming systems. Effective control and preventive measures can be applied once the risk factors are identified.

## Methods

### Study sites

**Fig. 1:**
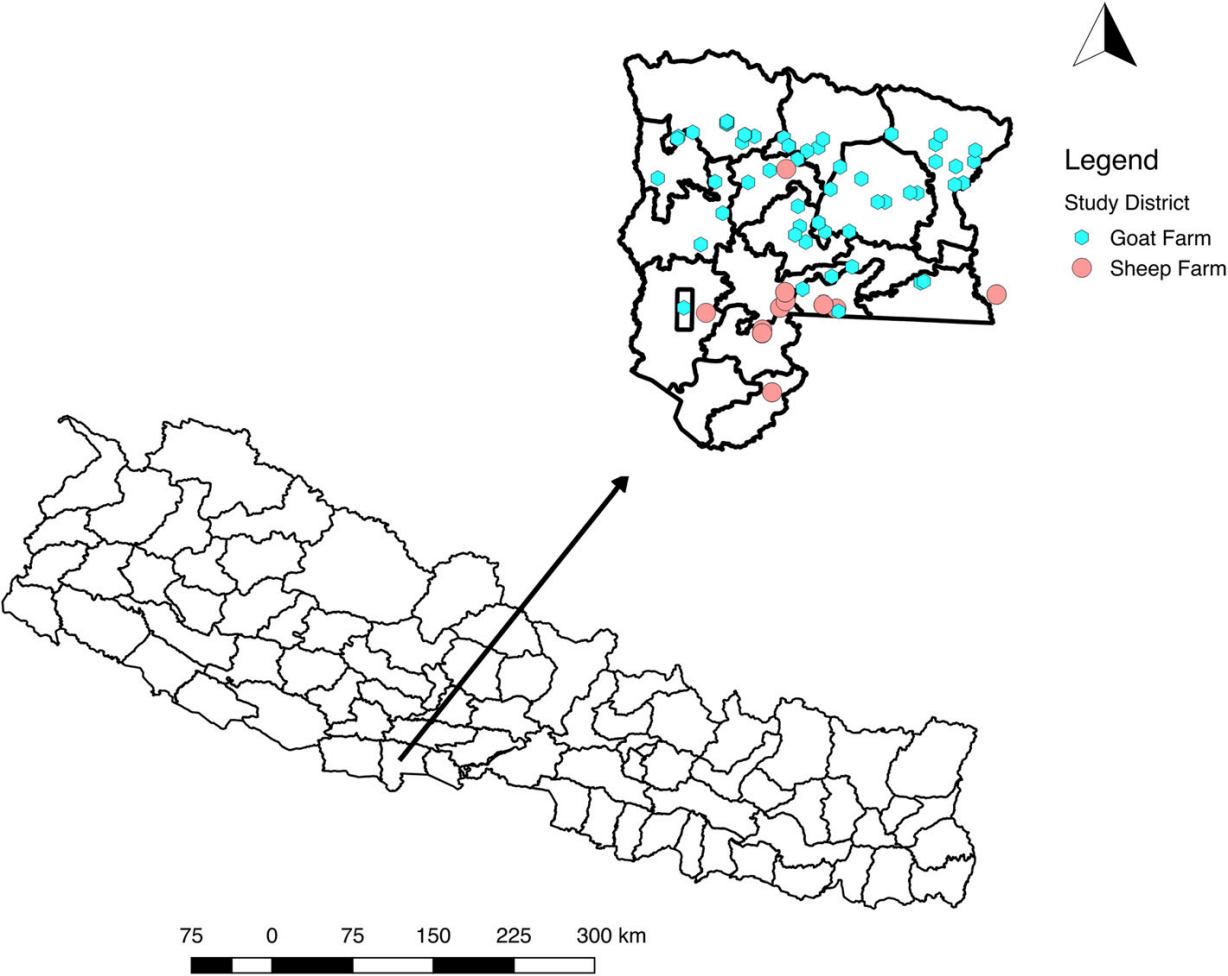
A map of Nepal with the study district indicated, and locations of sheep and goat farms in it (generated using QGIS 2.18)

This study was conducted in Rupandehi district in the southwestern region of Nepal (Fig. 1, created using QGIS). According to Livestock Statistics Report of Nepal (2017), the population of goat and sheep in Rupandehi were 185,332 and 4024, respectively [16]. This district was selected for study because it shares the border with northern India from where goats are imported into Nepal [27]. This brings an increased risk of disease introduction into the small ruminant population in Nepal. Informal livestock trade can even be more detrimental when the risk of disease introduction is concerned. Also, there is a risk of disease spread due to the internal movement of goats and sheep between other adjacent districts.

### Study design

A cross-sectional study was conducted on the goat and sheep population of Rupandehi district between January to March 2020. Semi-structured questionnaire was administered to collect the information on each flock’s animal characteristics, management status, and animal movement system. The survey was initially designed in English and later translated to the local Nepali language. Next, blood samples were collected during the time of interviewing the sheep and goat herd owners. Written consent from the owners was obtained during the questionnaire and sample collection processes.

### Sampling and sample size calculation

A sampling frame was constructed to list all the registered goat and sheep farms in the district. There are 16 local levels in the Rupandehi district with 106 accessible commercial goat and sheep flocks [28]. The total number of the flock was calculated by assuming that the prevalence of the disease in the congregation was 50% at 95% confidence interval (CI) with 5% desired precision by using the following formula (Eq. 1).

The formula is based on:

1 $$ \mathrm{N}\kern0.5em =\frac{\kern0.5em 1.{96}^2{\mathrm{P}}_{\mathrm{exp}}\left(1-{\mathrm{P}}_{\mathrm{exp}}\right)}{{\mathrm{d}}^2} $$

where *N* = sample size, P_exp_ = expected prevalence, and d = absolute precision [29].

The total number (N) of the flocks to be selected by this method was 84.

A total of 84 farms in the district were selected, but five farms did not agree to participate. Thus, samples were collected from 79 farms (60 goat farms and 19 sheep farms) only. In total, 357 sera samples (277 goat and 80 sheep samples) were collected for this study. The number of animals selected from each flock was based on their herd size. At least 5 % of animals from each flock were sampled for the study.

### Laboratory analysis

The collected samples were stored in an ice pack and transported to the laboratory for further analysis. A Rose Bengal plate test was performed as a screening test for brucellosis. All serum samples, including both Rose Bengal seropositive and seronegative samples, were tested by ELISA. All the tests were performed at Central Veterinary Laboratory (CVL), the national veterinary reference laboratory.

#### Rose Bengal test (RBT)

Rose Bengal Antigen (ID vet, France) was used as a rapid test to screen for antibody to *Brucella* spp. with a published sensitivity of 87.2% [30] and specificity of 99.6% [31]. The test serum (0.03 ml) was mixed with an equal volume of RBT antigen on a glass slide to produce a zone of approximately 2 cm in diameter. The mixture was agitated gently for 4 min at ambient temperature and then observed for agglutination. Tests were considered positive when any visible reaction or agglutination were observed.

#### ELISA (enzyme-linked immunosorbent assay)

The “ID Screen® Brucellosis Serum Indirect Multi-species” i-ELISA kit (ID vet France, kit reference BRUS-MS-5P) was used to test sera. The kit detects antibodies to various species of smooth lipopolysaccharide (S-LPS) expressing *Brucella*, such as *B. abortus, B. mellitensis,* and *B. suis*. The sensitivity and specificity of this test were 96.8 and 96.3%, respectively, according to the Bayesian estimation approach [32]. All the testing procedures were performed according to the protocols provided by the manufacturer. The test plates were read under the ELISA reader (“Multiskan™ FC Microplate Photometer”) at an optical density (OD) 450 nm within 15 min.

### Data management and statistical analysis

The raw data collected from the paper-based questionnaire was manually entered on the MS Excel spreadsheet and converted to CSV files. The data was analyzed using open-source epidemiological software Open epi and R version 3.6.1 [33]. The descriptive data analysis was performed to investigate the population characteristics of both the species.

Sheep and goat individual and flock-level prevalence were calculated to estimate the overall species-wise disease prevalence in the district. The RBT and ELISA were interpreted in parallel such that an individual or flock was considered seropositive if they tested positive to either test. For instance, an animal tested positive by either RBT or i-ELISA was considered a positive animal. Any herd with at least an animal tested seropositive to either RBT or I-ELISA was deemed positive flock. The chi-square test of associations and inter-test agreement between the Rose Bengal Test and ELISA tests for the seropositivity of brucellosis for sheep and goat were calculated [34, 35].

The Q-Q plot for the normality assessed the data distributions of each continuous variable. These continuous variables, such as flock size, age, and parity number, were converted into binary categorical variables using the quartile of distributions (e.g., median) to manage the problem of linearity [36].

Each of the independent factors was examined with the response variable (seropositivity) using contingency tables. Fisher’s exact tests and chi-square tests (when appropriate) were used to examine associations between response variables and explanatory variables.

The empty or zero cells in the two by two table analyses were corrected by data modification methods by Argesti (2002) in Open epi to calculate corresponding odds ratios and *p* values [37–39]. In this case, to rectify the effect of small-sample bias on maximum likelihood estimation of the logistic model, firth logistic regression was used by “logistif” function in R and firth logit option in STATA in multivariable regression analysis [37, 38, 40–43].

Chi-square test of association between categorical variables, such as median flock size, median age category and median parity category, were performed. As there was a significant association (*χ*2 = 116.78, *p* < 0.001) between the age and parity category was noted, the parity category was dropped from the model because age was a biologically plausible risk factor [44]. The variable’s potential confounding effects were checked with the changes in the point estimates of the variables that remain in the model [34, 45]. Any changes in the coefficient with > 20% were included in the final model.

The potential risk factors with the significance level *p* ≤ 0.2 following the bivariate analysis were manually entered in the final multivariable model [34]. A backward stepwise variable selection was used to add the variable with the lowest *p*-value to construct a final model with a significance level of *p* ≤ 0.05. Any variables with *p*-value < 0.05 at multivariable logistic regression analysis were considered statistically significant risk factors. The above process was performed separately for each set of risk factors of goat and sheep flock for the valid comparison. The fitness of the final model was assessed by Hosmer- Lemeshow goodness of fit test, and the model was fit (*p* > 0.05).

## Results

### Descriptive study of animal population

The study involved 60 (75.94%) goat farms and 19 (24.1%) sheep farms; from which, a total of 277 (77.59%) goat samples and 80 (22.41%) sheep blood samples were collected and tested for brucellosis. The median ages of goat and sheep populations were 1.5 years each, while the mean ages of goat and sheep population were 2.1 ± 1.1 and 1.9 ± 1.7 years, respectively. The median flock sizes of the goat and sheep farms were 48 and 100, while the mean flock sizes were 66 ± 11, 93 ± 4, respectively. The characteristics of goat and sheep population are depicted in Table 1.

**Table 1:** Comparison of population characteristics for studied goat and sheep population

		Descriptive statistics					
**Species**	**Variables**	**Mean ± SE**	**Min.**	**Q1**	**Median**	**Q3**	**Max.**
**Sheep (*n*** **= 80)**	Age (years)	1.9 ± 1.7	0.2	1	1.5	2.5	8
	Flock size	93 ± 4	12	65	100	120	150
**Goat (*n*** **= 277)**	Age (years)	2.1 ± 1.1	0.2	1	2	2.5	13
	Flock size	66 ± 11	20	30	48	60	550

From the survey, 15% (12/80) of sheep were either purchased from nearby herds or brought from India, while 37.6% (104/277) of the goats were either collected from neighbouring districts or brought from abroad. About 90 % of the goat herds included in the study were registered farms, but more than half of the sheep herds were not registered. Interestingly, an indigenous community mainly residing at the terai belt of south western Nepal maintained most sheep flocks, which was the primary means of their livelihoods.

### Seroprevalence of brucellosis in goat and sheep population

The flock level prevalence for sheep and goat farms was 30% (6/19; 95% CI: 12, 54%) and 3.33% (2/60, 95% CI: 0.92, 11.36%) respectively. Of the total of 80 sheep samples tested, 12 (15%; 95% CI: 8.79–24.41), and among 277 goat samples tested 3 (1.1%; 95% CI: 0.37–3.14) were seropositive to *Brucella* spp. (Table 2). There is a significant difference between the proportion of sheep and goat populations with seropositivity to brucellosis (χ2 = 29.78, *p* < 0.001).

**Table 2:** Comparison of seroprevalence of *Brucella* among goats and sheep by sex and breed-wise classification

Variables	Category	Total number (%)	RBT positive (%)	ELISA positive (%)	Overall Prevalence (95%CI)
**Species**	**Sex**				
Goat	Male	65 (23.46)	0.00	0.00	1.1% (0.37–3.14)
	Female	212 (76.53)	2.36% (5/212)	1.42% (3/212)	
Sheep	Male	16 (20)	18.75% (3/16)	18.75% (3/16)	15% (8.79–24.41)
	Female	64 (80)	12.5% (8/64)	14.1% (9/64)	
	**Breeds**				
Goat*	Local	135 (48.74)	2.22% (3/135)	2.22% (3/135)	1.1% (0.37–3.14)
	Exotic	142 (51.26)	1.41% (2/142)	0.00	
Sheep	Lampuchhre	75 (93.75)	14.67% (11/75)	16% (12/75)	15% (8.79–24.41)
	Baruwal	5 (6.25)	0.0	0.00	

* Exotic breeds of goat included Boer and Jamunapari. Local breeds of goat included Khari and Terai

Antibodies to *Brucella* were detected only in female goats, but in the sheep populations, a higher proportion of males, 18.75% (3/16), were seropositive to *Brucella* than females, 14.1% (9/64). Only the local goat breeds, such as Khari, were seropositive to *Brucella* by ELISA. Lampuchhre is an indigenous sheep breed that had the highest burden of disease. The detailed illustrations of the sex-wise and breed-wise comparison of seroprevalence of *Brucella* among goats and sheep by both RBT and ELISA are described in Table 2.

There was a significant association (*χ*^2^ = 28.29, *p* < 0.001) between seropositivity of RBT and ELISA tests for brucellosis in sheep and goats [34] and an extremely high level of agreement between the tests (κ = 0.95, 95% CI: 0.85–1, *p* < 0.001) [35].

### Univariable regression analysis

The bivariate analysis of the sheep and goat data was depicted in Tables 3 and 4, respectively. Sheep greater than 1.5 years of age had significantly higher odds of brucellosis (OR = 4.29, 95%CI: 1.16, 20.63, *p* = 0.0406) than the sheep of age ≤ 1.5 years. There were significantly higher odds of brucellosis among sheep when flock size was > 100 (OR = 4.2, 95% CI: 1.19,15.91, *p* = 0.026) than the sheep herds of ≤100. Sheep that had parity greater than one were 4.11 more likely to be detected with brucellosis compared to sheep ≤1, but the result was statistically borderline significant (OR = 4.11, 95%CI: 0.98, 21.29, *p* = 0.055) (Table 3).

**Table 3:** Univariable analysis results of potential risk factors associated with sero-positivity of sheep population against *Brucella* spp.

Determinants	Total no. of sheep	***Brucella*** positive	***Brucella*** negative	Odds ratio (OR)	95% CI	***P*** value
**Animal Origin**						
Purchased	12	3	9	2.19	(0.85, 2.21)	0.32
Homebred	68	9	59	Ref		
**Age (median = 1.5 years)**						
> 1.5	37	9	28	4.29	(1.16, 20.63)	0.041*
≤ 1.5	43	3	40	Ref		
**Herd size (median = 100)**						
> 100	24	7	17	4.2	(1.19,15.91)	0.026*
≤ 100	56	5	51	Ref		
**Parity (median = 1)**						
> 1	24	6	18	4.11	(0.98,21.29)	0.055
≤ 1	40	3	37	Ref		
**Gender**						
Male	16	3	13	1.41	(0.28,5.53)	0.646
Female	64	9	55	Ref		
**Grazing**						
Yes	74	12	62	2.6	(0.12, 49.16)	0.154
No	6	0	6	Ref		
**Repeat breeding**						
Yes	11	3	8	2.94	(0.62,2.63)	0.199
No	53	6	47	Ref		

*P value< 0.05 means statistically significant

**Table 4:** Univariable analysis results of potential risk factors associated with sero-positivity of goat population against *Brucella* spp.

Determinants	Total no of goats	***Brucella*** Positive	***Brucella*** Negative	Odds Ratios (OR)	95% CI	***P*** value
**Animal Origin**						
Purchased	105	2	103	1.2	0.11, 26.11	0.87
Homebred	172	1	171	Ref		
**Age (median = 2 years)**						
≤ 2	194	3	191	3.1	0.16, 59.74	0.12
> 2	83	0	83	Ref		
**Herd size (median = 48)**						
≤ 48	140	3	137	7	0.36, 136.8	0.06
> 48	137	0	137	Ref		
**Parity (median = 1)**						
≤ 1	113	3	110	6.5	0.33, 127.2	0.04*
> 1	102	0	102	Ref		
**Gender**						
Female	211	3	209	2.19	0.112, 42.9	0.34
Male	65	0	65	ref		
**Grazing**						
Yes	92	3	89	14.5	1.1, 283.9	0.01*
No	185	0	185	Ref		
**Repeat breeding**						
Yes	42	1	41	2.1	0.18, 23.28	0.29
No	171	2	169	Ref		

*P value< 0.05 means statistically significant

In bivariate analysis, the only variable associated with seropositivity was that the goats taken for grazing had significantly higher odds (OR = 14.5, 95% CI: 1.1, 283.9, *p* = 0.003) of *Brucella* seropositivity compared to goats stall-fed at farms (Table 4).

### Multivariable logistic regression analysis

The variables that qualified from the sheep data for multivariable analysis (*p* < 0.20) were age, gender, grazing system and disinfection process applied at the farm entry point. Similarly, for the goat data, the same sets of the variables were qualified for final firth multivariable logistic regression analysis based on the cut-off criteria of *p* < 0.20.

In the multivariable regression analysis, sheep of older age (> 1.5 years) had significantly higher odds (OR = 5.56, 95% CI: 11.39, 29.38, *p* = 0.02) of *Brucella* seropositivity compared to the younger sheep (≤1.5 years) (Table 5). The sheep farms of flock size greater than 100, had higher odds (OR = 4.74, 95% CI: 1.23, 20.32, *p* = 0.03) of *Brucella* seropositivity than those of smaller farm size.

**Table 5:** Multivariable analysis results of risk factors (*p* < 0.05) associated with sero-positivity of sheep population against *Brucella* spp.

Determinants	Category	Coefficient	Standard Error	Odds ratio (OR	95% CI	***P*** value
Age (median = 1.5 years)	> 1.5	1.72	0.76	5.56	(1.39, 29.38)	0.02*
	≤1.5					
Herd size (median = 100)	> 100	1.56	0.70	4.74	(1.23, 20.32)	0.03*
	≤100					

*P value < 0.05 means statistically significant

In the goat population, none of the variables was identified as statistically significant (*p* < 0.05) risk factors for brucellosis after running multivariable firth logistic regression (Table 6). Goats from the frequent grazing herds had higher odds (OR = 13.82, 95% CI: 0.70, 272.20) of *Brucella* seropositivity than the goats from isolated herds (Table 6), but this was borderline statistically significant (*p* = 0.08).

**Table 6:** Multivariable analysis results of risk factors (*p* < 0.05) associated with sero-positivity of goat population against *Brucella* spp.

Determinants	Category	Coefficient	Standard Error	Odds ratio (OR)	95% CI	***P*** value
Age (median = 2 years)	> 2	0.98	1.54	2.24	(0.12, 45.89)	0.60
	≤2					
Herd size (median = 48)	> 48	1.86	1.53	6.44	(0.32, 128.16)	0.22
	≤48					
Grazing	Yes	2.63	1.52	13.82	(0.70, 272.20)	0.08 ^a^
	No					

^a^: This variable has borderline significant p-value and could be a potential risk factor

## Discussion

### Seroprevalence of brucellosis between sheep and goats

We conducted a comparative study on the epidemiology of *Brucella* among goat and sheep herds in Rupandehi district. The burden of brucellosis was higher among the sheep, 15% (95% CI: 8.79–24.41), compared to goats, 1.1% (95% CI: 0.37–3.14). A seasonal study [46] estimated that 6.6% (*n* = 212) of sheep and 3.4% (*n* = 774) of goats from various districts of Nepal were seropositive by indirect multispecies ELISA. It also determined that the prevalence of brucellosis was higher in sheep than goats which supports the findings of the current study. This variation in seroprevalence between small ruminant species might be due to differences in the herding practices. In Nepal, a transhumant rotational sheep grazing system is used in many parts of the country. The seasonal migratory pattern of sheep could contribute to a higher rate of transmission in these animals [14]. This finding was also supported by a study conducted [44] in Tajikistan.

It has been suggested that goats are more susceptible to *B. melitensis* infection than sheep [47], but this might also reflect differences in the variation in geographical settings and differences between management of livestock production systems for sheep and goats. There were low numbers of seropositive goats in our research (3/227); however, if we had conducted our study from September to October, the prevalence of the disease would likely increase, due to the higher number of animal movements around this time for the ritual slaughter in Nepal. As none of the sheep and the goat flocks were vaccinated, the result of this study was evidence of natural infection transmission of brucellosis within the small ruminants in the study areas.

The prevalence of brucellosis in female sheep and goats were higher compared to that in males. This might be because the female sheep and goats will remain in the herd for a longer time as they are generally not slaughtered for meat, but retained for breeding in Nepal. Females are sold or exchanged between the flocks for kidding and they might have a higher risk for exposure to *Brucella* infected animals in the new environment, or they might bring the infection with them. Biologically, females are highly susceptible to *Brucella* spp. due to presence of erythritol in their gravid uterus [48].

### Significant risk factors

This work describes the first brucellosis associated risk factors study in small ruminants in Nepal to the best of our knowledge. Brucellosis is one of the priority zoonoses of the Government of Nepal. There might be some differences between the local risk factors identified with those identified elsewhere, but effective disease management lies in localized ways of managing the diseases.

The sheep population of age greater than 1.5 years had significantly higher odds of *Brucella* seropositivity than the younger ones. It might be because the older sheep remained in the flock for a long time, and they had a longer duration of exposure [44]. It is supported by many other studies [44, 49] that reported that biologically younger animals were more resistant to infection than adult animals. Nevertheless, as we could not detect active infection status of brucellosis in sheep either by bacterial isolation or use of polymerase chain reaction (PCR) in this study, the actual age-related risk factor was not determined.

Interestingly, age was not a significant risk factor for brucellosis in the goat population in the district. This may be because goat flocks were mainly maintained for meat production in Nepal, and most animals were sent to slaughter within a year.

The larger flock size (> 100) of sheep was another significant (*p* = 0.03) risk factor for brucellosis in this study. It is likely that the risk of disease transmission increased as the flock of sheep moved around in a large number of activities such as grazing. Also, the sheep flocks we visited were managed closer to one another such that the transmission of diseases between herds was more likely as they could mix up during grazing.

None of the variables related to the goats was significantly associated with *Brucella* antibody detection in the multivariable analysis. It might be due to the low prevalence of seropositive animals (*n* = 3/277) among goat population in the district. However, the difference in seroprevalence between grazing goats and stall-fed goats warrants further investigation. An additional complicating factor may be that while sheep and goat flocks were managed in geographically distinct areas, there was a higher probability of intermingling when taken for grazing in the pasture, watering points or animals moved to live markets.

Like other studies, our study had some limitations. The complement fixation test (CFT) for confirmation of brucellosis in sheep and goat populations was not performed as it was not available in the Central Veterinary Laboratory. Furthermore, *B. ovis* might be present in tested samples, but the diagnostic tests applied would not have picked it up due to low cross-reactivity of smooth LPS *Brucella* spp. Another limitation is that we could not address a detailed examination of the farmworker’s occupational safety issues in this study.

The findings from this work provide better epidemiological insight that could be utilized to improve management of important diseases such as brucellosis in small ruminants in Nepal. Discovering such a substantial burden of brucellosis in small ruminants, mostly in sheep, asks for the development of more focused control strategies by the Department of Livestock Services (DLS), Nepal.

## Conclusion

The estimation of disease burden and identifying risk factors associated with seropositivity of brucellosis in sheep and goat suggests that this disease is not evenly spread among small ruminants in Nepal. It is related to economic and occupational safety issues that needs to be considered when controlling brucellosis in Nepal. Increased age and larger flock sizes were the key risk factors for *Brucella* seropositivity among the sheep population. Grazing of goats may also be a risk factor in the goat population for *Brucella* seropositivity, and this needs to be investigated more thoroughly. Goats and sheep are valuable commodities for the livelihood of Nepalese farmers.

The prevention and control of brucellosis are crucial to the long term progress of this industry and for the safety of people who work with these animals. Brucellosis has been mentioned in the documents of the Government of Nepal as one of the priority zoonoses, but real-time disease surveillance and reporting are almost absent. This study could provide insights into the epidemiologic aspects of brucellosis in Nepal. However, we suggest further studies on the national level to get the bigger picture of animal brucellosis epidemiology in Nepal.

## Abbreviations

NVC: Nepal Veterinary Council
CVL: Central Veterinary Laboratory
RBT: Rose Bengal Agglutination Test
ELISA: Enzyme Linked Immunosorbent Assay
LPS: Lipopolysaccharide
OD: Optical Density
PC: Positive Control
NC: Negative Control
